# Semi-automated strain engineering and screening in the thermophilic fungus *Myceliophthora thermophila* for recombinant protein production

**DOI:** 10.1128/aem.00995-26

**Published:** 2026-08-28

**Authors:** Chaohui Gong, Xinyu Yang, Xiucai Zhao, Fufan Gou, Jie Zhou, Wenliang Sun, Tao Sun, Wenlong Liu, Kefen Wang, Xingji Wang, Yefu Chen, Yihan Liu, Chaoyou Xue, Qian Liu, Chaoguang Tian

**Affiliations:** 1 College of Biotechnology, Tianjin University of Science and Technology 66345, Tianjin, China; 2 State Key Laboratory of Engineering Biology for Low-Carbon Manufacturing, Tianjin Institute of Industrial Biotechnology, Chinese Academy of Sciences 165087, Tianjin, China; 3 National Technology Innovation Center of Synthetic Biology, Tianjin, China; 4 Longda Biotechnology Inc, Linyi, Shandong, China; Michigan State University, East Lansing, Michigan, USA

**Keywords:** *Myceliophthora thermophila*, filamentous fungi, recombinant protein production, semi-automated screening, thermophilic fungal chassis, activity-based screening

## Abstract

Thermophilic filamentous fungi are widely used for cellulase production at elevated temperatures; however, efficient recombinant protein production in these organisms remains limited because construction and screening of large transformant populations remain difficult. Here, *Myceliophthora thermophila* was engineered into a thermophilic fungal chassis through combinatorial deletion of genes associated with proteolysis, vesicle trafficking, and secretion, resulting in a hyper-secretory strain. An optimized expression cassette coupled with a laccase reporter enabled activity-based identification of productive transformants. Iterative genome engineering combined with QPix-assisted colony picking enabled parallel recovery and screening of fungal transformants, with 552 colonies recovered after transformation and dilution plating and 96 transformants further evaluated by ABTS-based plate screening. Targeted integration at the MtLacA locus enabled identification of productive recombinants through loss of laccase activity, and transformants frequently carried multicopy integrations. Through iterative screening and strain improvement, extracellular protein production reached ~1.1 g/L in shake-flask cultures and 5.9 g/L in a preliminary 5-L fermentation after 144 h using glucose as the carbon source. The chassis also supported the secretion of recombinant fungal enzymes and porcine growth factors. Collectively, these results establish a practical semi-automated workflow for recombinant protein production and strain engineering in thermophilic filamentous fungi.

**IMPORTANCE:**

Filamentous fungi are important industrial hosts, but strain engineering in these organisms is often constrained by low transformation efficiency, labor-intensive colony isolation, and limited screening throughput. In this study, we established a semi-automated engineering workflow in the thermophilic fungus *Myceliophthora thermophila* that combines dilution-based colony recovery, QPix-assisted colony isolation, and activity-based screening. More than 500 fungal colonies could be recovered and processed during a single engineering round, allowing rapid evaluation of transformants by ABTS-based plate screening. In addition to enhanced laccase production, the engineered chassis supported the secretion of multiple heterologous proteins, including fungal enzymes and porcine growth factors. These findings demonstrate the potential of thermophilic filamentous fungi as recombinant protein production hosts and provide a practical workflow for improving strain engineering efficiency in filamentous fungal systems.

## INTRODUCTION

Filamentous fungi are widely used as industrial hosts for the production of enzymes, proteins, organic acids, biofuels, and other biochemicals (1, 2). Their strong capacity for extracellular protein secretion and growth on plant-derived substrates has supported extensive strain development in genera such as *Trichoderma*, *Aspergillus*, and *Penicillium* (3–5). Fungal cellulases and amylases are widely used in lignocellulosic biomass conversion and starch-processing industries, where enzymatic saccharification is important for production of sweeteners and bioethanol (6–8). Thermophilic filamentous fungi provide additional industrial advantages, including elevated growth temperatures, reduced cooling demand, lower contamination risk, and the production of thermostable enzymes that remain active under harsh processing conditions (9, 10). The thermophilic ascomycete *Myceliophthora thermophila* (synonym *Thermothelomyces thermophilus*) has attracted increasing attention because genome and multi-omics analyses have revealed a broad repertoire of carbohydrate-active enzymes involved in lignocellulose degradation (11–13). In addition, industrial strains derived from *Chrysosporium lucknowense* C1 can secrete enzyme mixtures at very high protein titers, highlighting the potential of thermophilic fungi as efficient enzyme production hosts (14).

Protein secretion in filamentous fungi is controlled by interconnected transcriptional, signaling, and nutrient-responsive pathways (15–17). Catabolite repression, substrate-responsive transcription factors, and secretory-pathway feedback collectively influence both enzyme production and extracellular protein composition (18, 19). Manipulation of regulators such as ACE4, MtTRC-1, and TAM1 has been shown to alter fungal secretomes and improve lignocellulose conversion phenotypes (20–22). Beyond native enzyme production, filamentous fungi have also been developed as hosts for heterologous protein expression. In *T. reesei*, marker-recycling CRISPR/Cas systems have enabled the construction of strains with reduced background secretion, facilitating production of recombinant enzymes and heterologous proteins (23, 24). Other studies have adopted screening-guided strain evolution strategies to improve protein production in filamentous fungi. For example, sub-genomic RNAi libraries combined with fluorescence-assisted cell sorting enabled iterative evolution of *T. reesei* for enhanced cellulase production, and a similar strategy was extended to *Humicola insolens* for catalase production (25). These studies also highlighted a persistent limitation in filamentous fungi: transformation efficiency is generally much lower than in bacterial or yeast systems. In parallel, *A. niger* has been developed as a GRAS production host, where promoter engineering, signal peptide optimization, and multicopy CRISPR/Cas9 integration have been used to improve recombinant enzyme secretion (26–30).

Recent advances in genetic engineering have substantially improved the tractability of *M. thermophila* as a thermophilic production host. CRISPR–Cas9 and Cas12a systems, 2A peptide–based expression strategies, and base-editing tools now enable multiplex genome engineering, coordinated gene expression, and marker recycling in this fungus (31–34). A flow cytometry-based, plating-free transformation workflow has further accelerated strain construction by enabling direct sorting of transformed protoplasts into deep-well plates (35). These developments have supported the use of *M. thermophila* for enzyme production and biosynthesis of bulk chemicals (36–38). Functional studies have also identified several regulatory and protease-related genes that restrict protein secretion, including *MtAlp1, MtSpr-14, Ap3m, Stk-12, Dsc-1, Dsc-2, Sah-2, and Exo-1*, providing targets for engineering improved secretory capacity (39–44).

Despite these advances, efficient construction and screening of large fungal transformant populations remain challenging. Filamentous fungi generally exhibit lower transformation efficiencies, slower colony development, and greater morphological heterogeneity than bacteria and yeast. Automated methods for colony recovery and phenotypic screening are also less established. As a result, iterative genome engineering still depends heavily on manual handling. In *M. thermophila*, previous heterologous expression studies have mainly focused on the optimization of promoters, 5′ UTRs, and signal peptides in relatively simple genetic backgrounds (45). Strategies that combine multigene chassis engineering with parallel transformant handling and activity-based screening remain limited.

Here, we developed a thermophilic fungal chassis and a semi-automated screening workflow for recombinant protein production in *M. thermophila*. The chassis strain GM8 was constructed through successive deletion of eight regulatory and protease-related genes. It showed increased extracellular protein secretion on Avicel, starch, and glucose. We then combined optimized expression elements with an ABTS-based colorimetric assay to screen laccase-producing transformants. Marker-recycling genome editing and QPix-assisted colony picking enabled parallel handling of fungal colonies after transformation. In a single engineering round, 553 colonies were recovered and subjected to resistance verification, and 96 transformants were further evaluated by ABTS-based plate screening. The resulting M10-OEMtLacA strain produced approximately 1.1 g L⁻¹ extracellular protein in shake-flask culture and 5.9 g L⁻¹ in a preliminary 5-L fermentation. The chassis also supported the secretion of fungal enzymes and porcine growth factors. These results demonstrate the utility of the workflow for recombinant protein production and iterative strain engineering in *M. thermophila*.

## MATERIALS AND METHODS

### Strains, media, and growth conditions

The *Myceliophthora thermophila* wild-type strain ATCC 42464 (MtWT) was obtained from the American Type Culture Collection (ATCC). *M. thermophila* strains were grown on Vogel’s minimal medium (MM) supplemented with 2% sucrose at 45°C for 7 days to obtain conidia. G418 or phosphinothricin was added when required for transformant selection. In shake-flask cultures, *M. thermophila* conidia were inoculated at 2.5 × 10⁵ spores mL⁻¹ into MM supplemented with 2% (wt/vol) Avicel, 2% (wt/vol) starch, or 4% (wt/vol) glucose as the sole carbon source and 0.5% (wt/vol) yeast extract and incubated at 45°C with shaking at 150 rpm. For recombinant protein production, conidia from *M. thermophila* transformants were inoculated into 50 mL of MM containing 4% (wt/vol) glucose and 0.75% (wt/vol) soybean meal in 250-mL Erlenmeyer flasks and cultivated at 45°C with shaking at 150 rpm. For preliminary batch fermentation, cultivations were performed with 3 L medium in 5-L fermenters (Baoxing Biological Engineering Co. Ltd., China), as previously described (36). For vector construction, *Escherichia coli* Trans-T1 cells (TransGen, Beijing, China) were cultured in LB medium supplemented with kanamycin (50 mg L⁻¹) or ampicillin (100 mg L⁻¹), as required.

### Vector construction for genome engineering and recombinant protein expression

All primers used in this study are listed in Table S1. PCR amplification was performed using Super-Fidelity DNA Polymerase (Vazyme, Nanjing, China). Plasmids were assembled using the Gibson Assembly Cloning Kit (New England Biolabs, Beijing, China). For genome engineering, single-guide RNA (sgRNA) expression cassettes and donor DNA constructs were prepared as described previously (31, 32). The target genes included *Mtalp-1* (Mycth_2303011), *Mtspr-14* (Mycth_2304704), *Mtexo-1* (Mycth_2296508), *Mtap3m* (Mycth_2307451), *Mtstk-12* (Mycth_2297068), *Mtdsc-1* (Mycth_2300902), *Mtdsc-2* (Mycth_2311528), and *Mtsah-2* (Mycth_2306768). Two selectable markers, *neo* (GenBank: HQ416708) and *bar* (GenBank: X17220), were used where indicated. For each target locus, the 5′ and 3′ flanking regions were amplified from the *M. thermophila* genome and assembled with a selectable marker cassette (PtrpC-neo or PtrpC-bar) (32) into pUC118.

For recombinant protein expression, coding sequences encoding *M. thermophila* laccase (MtLac1, NCBI: XP_003663741.1), *Talaromyces emersonii* glucoamylase (TeGlaA, UniProt: Q9C1V4), *A. niger* glucose oxidase (AnGoxM, NCBI: MK071618.1), porcine epidermal growth factor (EGF, NCBI: AIS22039.1), and porcine transforming growth factor beta-1 proprotein (TGFβ1, NCBI: NP_999180.2) were used. Expression cassettes were assembled by combining the *M. thermophila* Ppdc promoter (from Mtpdc, Mycth_112121), the synthetic 5′-UTR NCA-7d (45), the native signal peptide of MtLac1, the target coding sequence, and the MtLac1 terminator (Tlac1) into pAN52-hph (35). All coding sequences encoding heterologous proteins were codon-optimized for expression in *M. thermophila* and synthesized by Genewiz (Suzhou, China). An N-terminal 6× His tag followed by an enterokinase recognition site (DDDDK) was placed immediately upstream of each coding sequence.

### Construction of *M. thermophila* strains

Genetic manipulation of *M. thermophila* was performed using a modified protoplast-mediated transformation protocol combined with a CRISPR/Cas9-based marker-recycling strategy, as described previously (31, 32). Transformants were cultured on Vogel’s MM agar plates and incubated at 37°C for 4–5 days under selection with either G418 (100 mg L⁻¹) or phosphinothricin (200 mg L⁻¹). Correct gene disruptions were verified by PCR analysis.

For recombinant strain construction, an automated workflow was implemented to reduce manual handling during transformant isolation and screening. Liquid handling was used for medium preparation and dispensing, and colonies were isolated using an automated colony-picking system (QPix 420, Molecular Devices), enabling parallel recovery and handling of fungal transformants after dilution plating. Individual colonies were transferred onto ABTS-containing selective plates for rapid phenotypic screening. Active laccase secretion was identified by the appearance of dark green coloration surrounding colonies due to ABTS oxidation.

For heterologous protein expression, target genes were integrated into the MtLacA expression locus, replacing the laccase expression cassette. Transformants lacking ABTS oxidation activity were selected as candidate recombinants, and high-producing strains were subsequently identified by downstream protein and activity analyses. Selected transformants were cultivated in shake flasks, and recombinant protein expression was confirmed by SDS–PAGE, enzyme activity assays, and Western blot analysis. The copy number of integrated expression cassettes was determined by quantitative PCR (qPCR) following a previously reported method (35).

### Protein quantification, SDS-PAGE, and Western blot analysis

Extracellular protein concentrations were determined using the Bio-Rad DC Protein Assay Kit according to the manufacturer’s instructions (Bio-Rad, USA). SDS-PAGE was performed using polyacrylamide gels (Thermo Fisher Scientific, USA). Fungal biomass was harvested, dried to constant weight, and weighed to determine the dry biomass. Protein levels were normalized to the average dry biomass weight. All experiments were conducted with three biological replicates.

For Western blot analysis, culture supernatants from recombinant strains cultivated in the glucose-containing medium for 3 days were collected and separated by SDS-PAGE. Proteins were electrotransferred onto PVDF membranes and blocked with 5% (wt/vol) skim milk in TBS buffer for 1 h at room temperature. The membranes were incubated overnight at 4°C with a rabbit anti-His primary antibody (Cat. No. AE086, Abclonal, Wuhan, China) diluted at 1:5,000 in TBS buffer. After washing, membranes were incubated for 1 h at room temperature with a horseradish peroxidase (HRP)-conjugated goat anti-rabbit IgG secondary antibody (Cat. No. AS014, Abclonal, Wuhan, China) diluted at 1:10,000. Protein signals were detected using SuperSignal West Pico PLUS Chemiluminescent Substrate (Thermo Scientific, Cat. No. 34580, USA) and visualized with a ChemiDoc MP Imaging System (Bio-Rad, USA).

### Enzyme activity assays

For enzyme activity assays, culture samples were centrifuged at 12,000 × *g* for 10 min at 4°C to remove fungal hyphae. Clarified supernatants were transferred to fresh tubes and filtered prior to analysis. Laccase activity was determined by monitoring the oxidation of 2,2′-azino-bis(3-ethylbenzothiazoline-6-sulfonic acid) using a commercial laccase activity assay kit (BC1630, Solarbio Life Sciences, Beijing, China). One unit of laccase activity was defined as the amount of the enzyme required to oxidize 1.0 nmol of ABTS per minute per milliliter of the culture supernatant. Glucoamylase activity was measured using the 3,5-dinitrosalicylic acid method as described previously (35). One unit of glucoamylase activity was defined as the amount of enzyme releasing 1 μmol of glucose per minute from starch, with the absorbance recorded at 540 nm. Glucose oxidase activity in culture supernatants was assayed using a commercial glucose oxidase activity kit (BC0690, Solarbio Life Sciences, Beijing, China). One unit of activity was defined as the amount of the enzyme catalyzing the formation of 1.0 nmol of oxidized o-dianisidine dihydrochloride per min.

### Protein purification

N-terminal 6× His-tagged recombinant proteins were harvested from culture supernatants after 3 days of shake-flask cultivation. Culture broths were clarified by centrifugation and filtration prior to purification. For affinity purification, 100 mL of the clarified supernatant was concentrated approximately twofold by ultrafiltration using a Vivaflow 20 system equipped with a 3-kDa molecular weight cutoff membrane (Sartorius Stedim Biotech, Germany) in binding buffer containing 20 mM sodium phosphate (pH 7.4), 500 mM NaCl, and 5 mM imidazole. Concentrated samples were loaded onto a 5-mL HisPur Ni–NTA affinity chromatography cartridge (Thermo Fisher Scientific, USA) connected to an ÄKTA Purifier 10 system (GE Healthcare, USA). After washing with binding buffer, the bound proteins were eluted using a linear imidazole gradient from 10 to 500 mM in the same buffer. Eluted fractions were collected and analyzed by SDS-PAGE.

### Whole-genome resequencing and copy number variation analysis

Genomic DNA was extracted from the parental strain GM8-OEMtLacA-M4 and the engineered strains T8, T9, and T64. Whole-genome resequencing was performed by Novogene Corporation (Tianjin, China). Sequencing libraries were constructed according to the manufacturer’s standard protocol and sequenced on the DNBSEQ-T7 platform. Clean reads were aligned to the *M. thermophila* ATCC 42464 reference genome using Burrows-Wheeler Aligner (BWA). Copy number variation was analyzed using CNVnator, and the normalized read depth of the *MtLacA* reference region was used to assess relative changes in *MtLacA* copy number.

### Statistical analysis

All experiments were performed with three independent biological replicates. Data are presented as mean ± standard deviation. Statistical significance was assessed by one-way analysis of variance, with differences considered significant at *P* < 0.01.

## RESULTS AND DISCUSSION

### Construction of a high-secretion chassis by multi-gene engineering

Filamentous fungi are widely used for industrial protein production because of their strong extracellular secretion capacity; however, abundant secretion of endogenous enzymes often complicates heterologous protein expression by increasing background protein levels (23, 29, 46). To develop a strain background with improved secretion capacity and reduced proteolytic interference, genes associated with extracellular proteolysis, vesicle trafficking, and secretion-related regulation were sequentially disrupted in *M. thermophila*.

**Fig 1:**
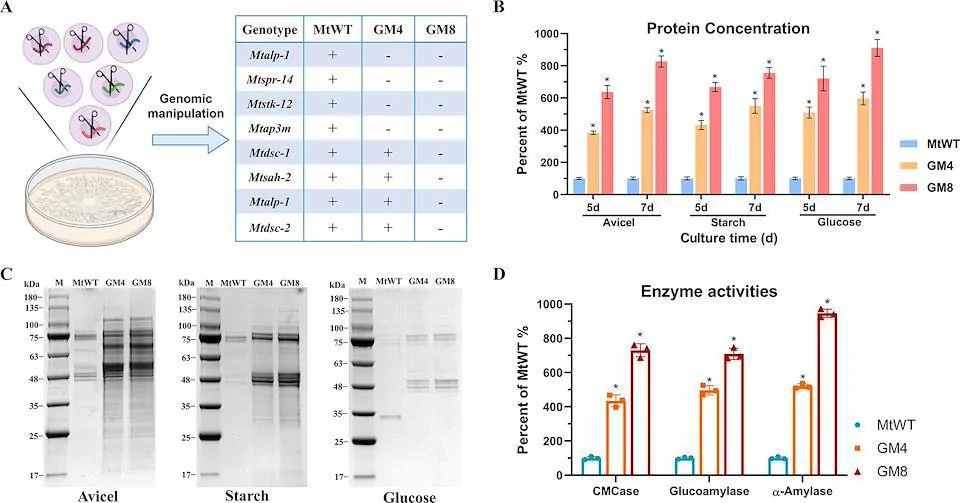
Construction and secretion phenotype analysis of the engineered *M. thermophila* strains MtWT, GM4, and GM8. (**A**) Schematic illustration of the stepwise genomic manipulations used to construct the strains GM4 and GM8 through iterative CRISPR/Cas-assisted marker recycling in this study. (**B**) Assays of total extracellular protein in culture supernatants of the *M. thermophila* strains after 5 and 7 days of cultivation on Avicel, starch, and glucose media. Protein concentrations are shown as percentages relative to MtWT. (**C**) SDS-PAGE analysis of the proteins secreted by the *M. thermophila* strains after cultivation on Avicel, starch, and glucose media. (**D**) Activities of CMCase, glucoamylase, and α-amylase in culture supernatants of the *M. thermophila* strains. Enzyme activities are shown as percentages relative to MtWT. Values are expressed as means ± SD (*n* = 3 biological replicates).

Based on previous studies (35, 39, 40, 43), four genes, *Mtexo-1, Mtstk-12, Mtspr-14*, and *Mtap3m*, were first deleted to generate the intermediate strain GM4 (Fig. S1). Subsequently, *Mtalp-1, Mtdsc-1, Mtdsc-2*, and *Mtsah-2* were further disrupted to construct the eight-gene mutant GM8 (Fig. 1A; Fig. S2). These iterative genome modifications were performed using the CRISPR/Cas-assisted marker recycling (Camr) strategy, enabling successive engineering in the same strain background (32, 43). Extracellular protein secretion by MtWT, GM4, and GM8 was compared after cultivation on Avicel, starch, and glucose for 5 and 7 days (Fig. 1B). Under all tested conditions, GM4 produced substantially more extracellular protein than the wild-type strain, whereas GM8 consistently showed the highest secretion levels. The difference became more pronounced after prolonged cultivation. After 7 days of cultivation, GM8 reached approximately 8-fold of the MtWT protein level in Avicel medium and nearly 9-fold in glucose medium. Similar trends were observed in starch cultures, indicating that the engineered strains maintained enhanced secretion capacity across different carbon sources.

SDS-PAGE analysis supported these measurements (Fig. 1C). Compared with MtWT, extracellular protein bands were markedly intensified in GM4 and further increased in GM8. Secreted protein profiles also differed depending on the carbon source. Avicel cultures showed enrichment of cellulolytic proteins, whereas stronger amylolytic protein bands were observed in starch and glucose cultures. These patterns further supported enhanced extracellular secretion in the engineered strains. Enzyme activities were further quantified using CMCase, glucoamylase, and α-amylase assays (Fig. 1D). Relative to MtWT, CMCase, glucoamylase, and α-amylase activities in GM8 increased by approximately 7.3-, 7.1-, and 9.5-fold, respectively. Collectively, these results indicate that iterative disruption of secretion-associated genes improved extracellular protein production in *M. thermophila*.

Previous studies from our group systematically evaluated several individual secretion-related gene deletions, providing insights into their relative contributions (35, 43). Under starch conditions, deletion of Mtsah-2 resulted in the largest increase in extracellular protein secretion among the tested single-gene mutants, whereas deletion of Mtstk-12 also significantly enhanced protein secretion and glucoamylase activity (43). These observations suggest that Mtsah-2 and Mtstk-12 are likely to be major contributors to the high-secretion phenotype of GM8. However, because GM8 contains multiple engineered deletions, the contribution of each individual mutation cannot be directly inferred from the present study. Future systematic analysis of intermediate deletion combinations will be required to define a minimal secretion chassis.

Notably, GM8 retained high extracellular protein production under glucose conditions, whereas the wild-type strain secreted only limited amounts of protein. Because glucose cultivation generates substantially lower endogenous enzyme backgrounds than Avicel or starch, this condition facilitates downstream detection of recombinant proteins. These characteristics support the use of GM8 as a chassis strain for heterologous protein expression.

### Expression of a fungal laccase in the GM8 chassis strain with ABTS-based screening

**Fig 2:**
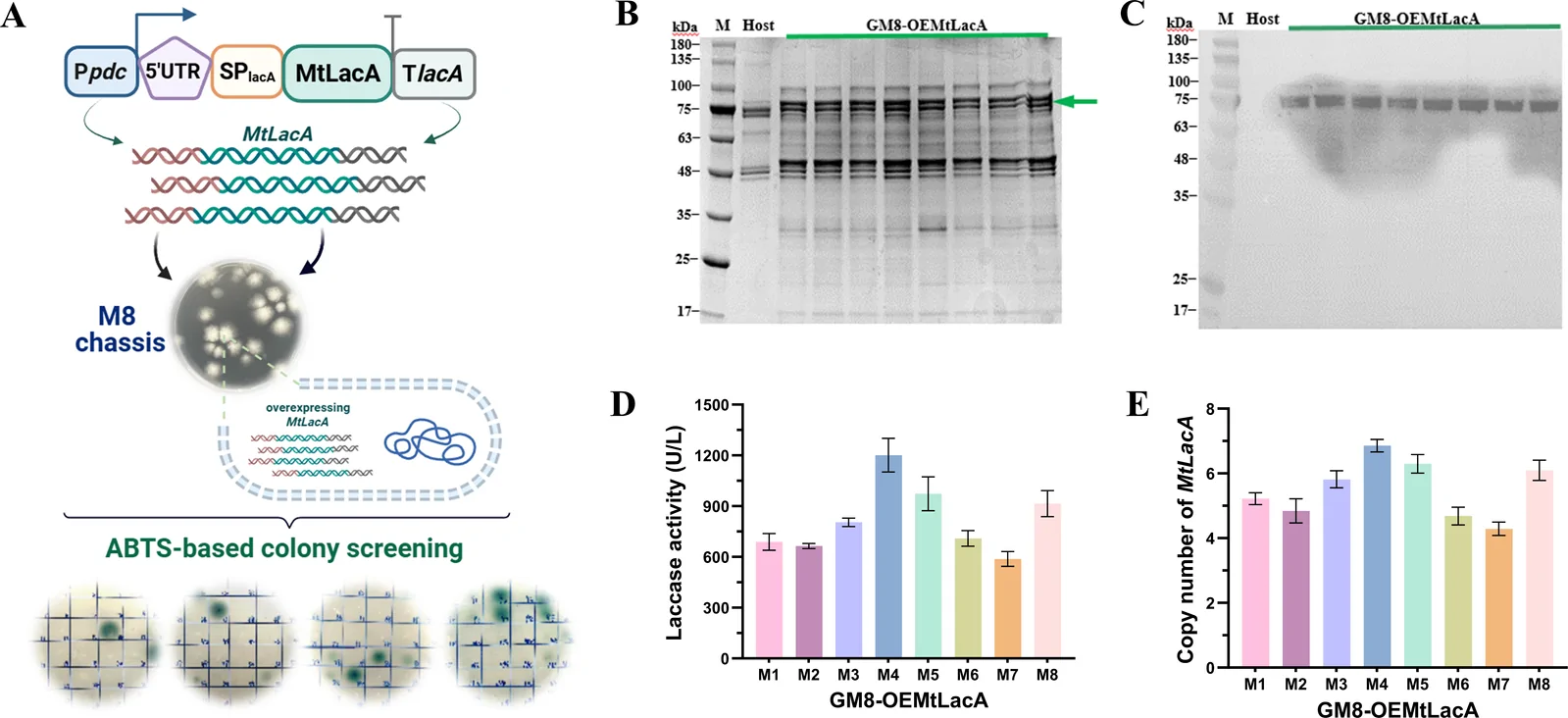
Construction and evaluation of the MtLacA overexpression chassis in the GM8 strain. (**A**) Schematic illustration of the MtLacA overexpression cassette and ABTS-based colony screening strategy. The cassette consists of a promoter (Ppdc), 5′-UTR, signal peptide (SPlacA), MtLacA coding sequence, and terminator (TlacA). Multicopy integration of MtLacA enables enhanced secretion and visual screening based on ABTS oxidation. (**B**) SDS-PAGE analysis of extracellular proteins from the host strain and representative GM8-OEMtLacA transformants. The arrow indicates the MtLacA band. (**C**) Western blot analysis confirming MtLacA expression in the engineered strains. (**D**) Laccase activity of GM8-OEMtLacA transformants (M1–M8) measured in shake-flask cultures. (**E**) Estimated copy number of the MtLacA expression cassette in representative transformants determined by qPCR. Error bars indicate standard deviation (SD) from three biological replicates.

To evaluate recombinant protein production in the engineered chassis, a homologous fungal laccase, MtLacA, was selected as an initial target protein. An expression cassette containing the constitutive promoter P*pdc*, a synthetic 5′-UTR (NCA-7d), and the native MtLacA signal peptide was constructed and introduced into GM8 (Fig. 2A). Transformants were screened on ABTS-containing plates, where colonies producing active laccase developed distinct green halos because of ABTS oxidation, whereas no coloration was observed in the host strain (Fig. 2A). This phenotype allowed rapid visual identification of productive transformants.

Selected transformants (GM8-OEMtLacA) were cultivated in glucose medium for extracellular protein analysis. Glucose medium was used to minimize interference from endogenous secreted proteins. SDS-PAGE analysis showed a prominent extracellular protein band at approximately 75 kDa in GM8-OEMtLacA strains, consistent with the expected molecular mass of MtLacA (45, 47), whereas the corresponding band was absent in the host strain (Fig. 2B). Western blot analysis further confirmed secretion of MtLacA in the engineered strains (Fig. 2C). Substantial variation in laccase activity was observed among transformants (Fig. 2D). Strain M4 showed the highest activity, reaching approximately 1,201 U/L, followed by M5 and M8. Quantitative PCR analysis showed that MtLacA copy numbers ranged from approximately 4 to 7 among the selected transformants (Fig. 2E). Transformants carrying higher copy numbers generally showed stronger laccase activities, although the relationship was not strictly proportional, suggesting that additional factors, including genomic position effects, also contributed to expression differences.

Collectively, these results confirmed that the engineered GM8 background supported the secretion of recombinant MtLacA. ABTS-based screening also provided a convenient phenotypic readout for identification of productive transformants.

### QPix-assisted screening and characterization of engineered MtLacA-producing strains

To further improve laccase production, the MtLacA expression cassette was reintroduced into the previously constructed strain GM8-OEMtLacA-M4 through an additional round of transformation and marker-recycling genome engineering. During this process, the selectable marker was switched from bar to neo, and the transcription factor gene *MtRes1* was deleted. Previous studies showed that deletion of the corresponding homolog influences lignocellulase secretion and fungal growth characteristics in filamentous fungi (31, 48, 49).

QPix-assisted colony isolation was incorporated into the workflow to handle the large number of transformants generated during iterative engineering (Fig. S3). In a single engineering round, 553 fungal colonies were recovered by dilution plating and processed using QPix. Of these, 96 transformants were selected for ABTS-based plate screening. Colonies with active laccase were readily identified by visible ABTS oxidation halos, although the halo intensity varied among transformants. Transformants showing successful marker turnover and detectable laccase activity were designated GM10-OEMtLacA. Representative transformants were then cultivated in shake flasks for protein analysis, with GM8-OEMtLacA-M4 used as the reference strain.

**Fig 3:**
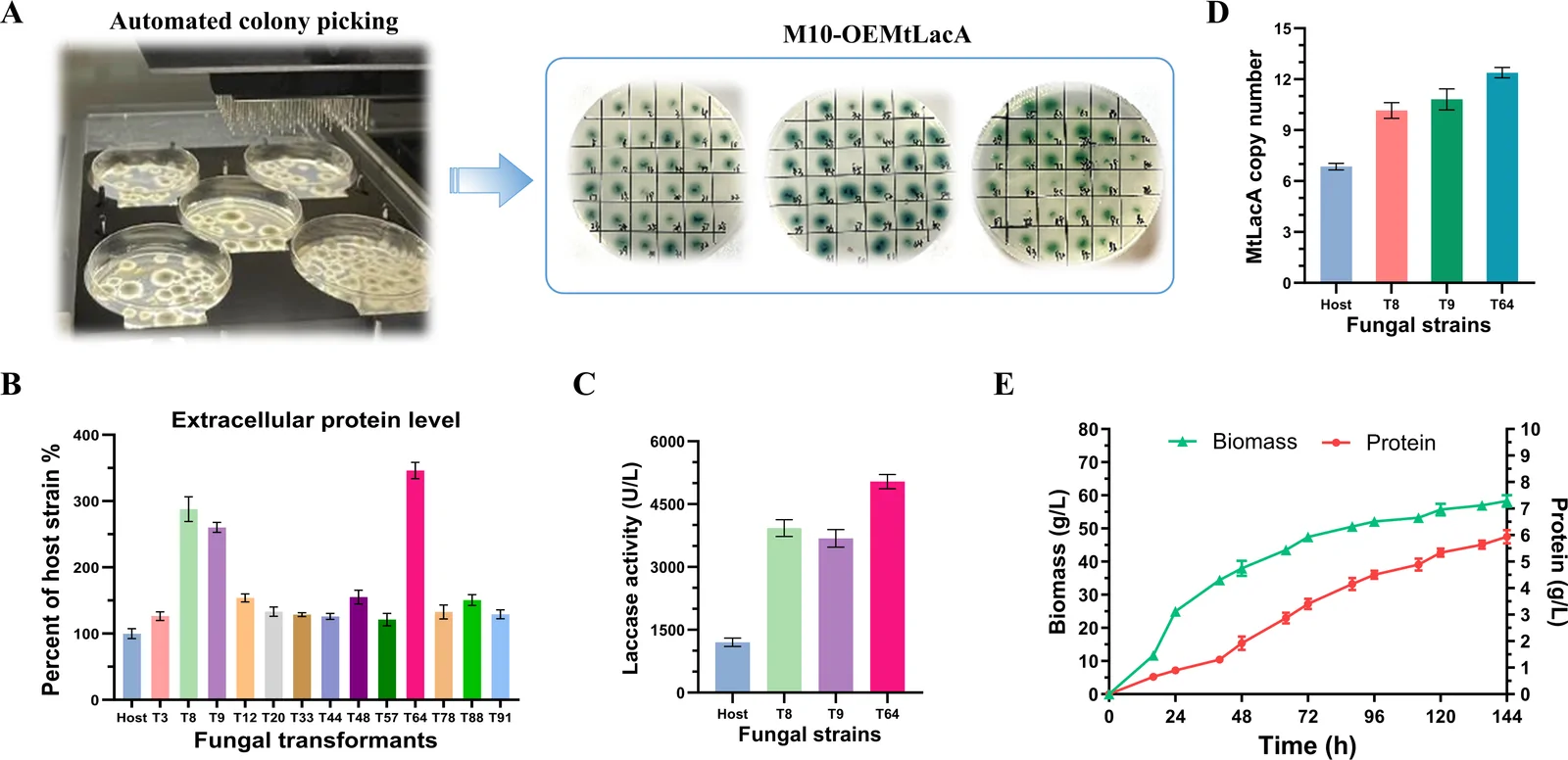
QPix-assisted screening and characterization of engineered MtLacA-overproducing strains. (**A**) Automated colony picking followed by ABTS-based screening of fungal transformants. Representative screening plates of the engineered M10-OEMtLacA strains are shown. (**B**) Relative extracellular protein levels of representative transformants cultivated in glucose-containing shake-flask medium, normalized to the host strain. (**C**) Laccase activities of selected M10-OEMtLacA transformants cultivated in shake-flask cultures. (**D**) Estimated copy number of the MtLacA expression cassette in the host strain and representative second-round transformants determined by qPCR. (**E**) Preliminary 5-L batch fermentation of strain T64 cultivated in glucose-containing medium for 144 h. Biomass accumulation and extracellular protein production were monitored at 24-h intervals during fermentation. Error bars indicate standard deviations from three biological replicates.

Representative transformants were subsequently cultivated in shake flasks for protein analysis, with GM8-OEMtLacA-M4 used as the reference strain. Extracellular protein levels varied considerably among transformants (Fig. 3B). Strain T64 showed the highest extracellular protein accumulation, reaching approximately 3.5-fold higher extracellular protein levels than GM8-OEMtLacA-M4, whereas strains T8 and T9 also showed increased extracellular protein accumulation. SDS-PAGE analysis further showed stronger extracellular protein bands in high-producing strains such as T8, T9, and T64 (Fig. S4).

Under glucose-based shake-flask cultivation, T64 reached approximately 1.1 g/L extracellular protein after 7 days. To further evaluate production performance, T64 was cultivated in a preliminary 5-L fermenter using glucose as the carbon source (Fig. 3E). Biomass accumulation increased rapidly during the early fermentation stage and approached a plateau after approximately 96 h, whereas extracellular protein accumulation continued throughout cultivation, reaching approximately 5.9 g/L at 144 h. Laccase activity also varied substantially among transformants (Fig. 3C). Compared with GM8-OEMtLacA-M4, T64 reached approximately 5,038 U/L, whereas T8 and T9 produced ~3,928 and ~3,681 U/L, respectively. Quantitative PCR analysis indicated that MtLacA copy number increased from approximately seven copies in GM8-OEMtLacA-M4 to 10–12 copies in the engineered strains (Fig. 3D). To further support the qPCR-based copy number estimation, whole-genome resequencing was performed for GM8-OEMtLacA-M4, T8, T9, and T64. Copy number variation analysis using CNVnator revealed a progressive increase in the normalized read depth of the MtLacA reference region (Fig. S6), consistent with the qPCR-estimated increase in MtLacA copy number. Although increased copy number was generally associated with higher laccase activity, the relationship was not strictly linear.

**Fig 4:**
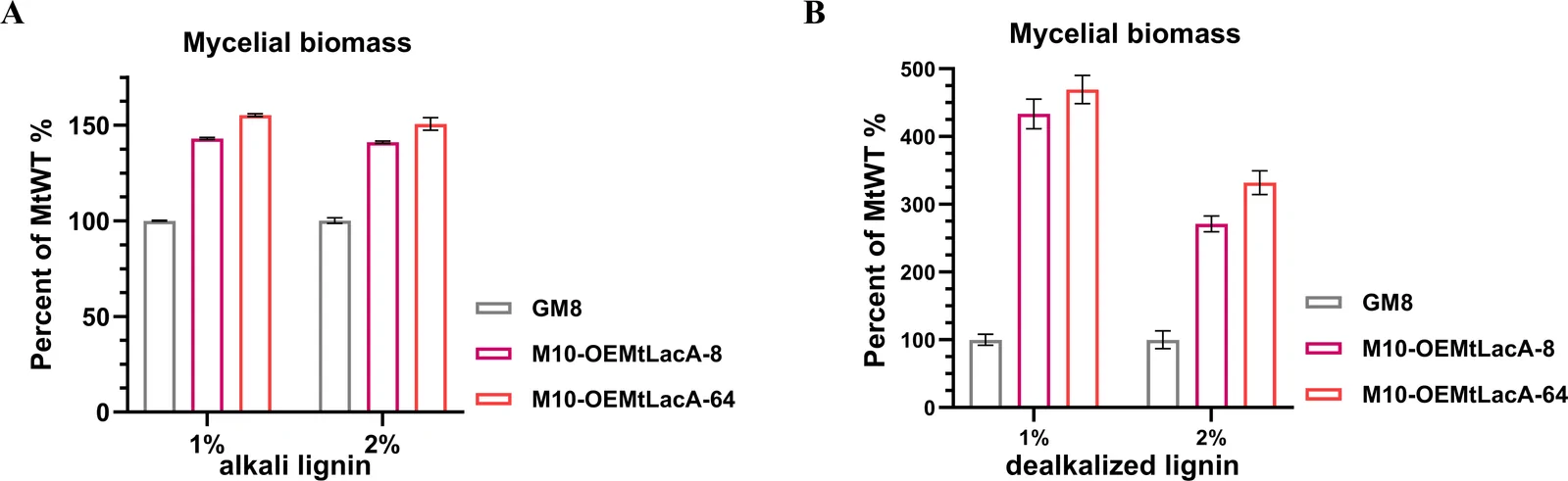
Effect of lignin on mycelial biomass of MtLacA-overexpressing strains. (**A**) Relative mycelial biomass of the GM8 strain and MtLacA-overexpressing strains (M10-OEMtLacA-8 and M10-OEMtLacA-64) in the presence of 1% and 2% alkali lignin. (**B**) Relative mycelial biomass of the same strains in the presence of 1% and 2% dealkalized lignin. Biomass is expressed as a percentage of the wild-type strain (MtWT). Error bars indicate the standard deviation (SD) from three biological replicates.

Growth on lignin-derived substrates was further evaluated (Fig. 4). Compared with GM8, both GM10-OEMtLacA-8 and GM10-OEMtLacA-64 exhibited increased biomass accumulation on alkali lignin and dealkalized lignin. The difference was particularly evident in dealkalized lignin cultures, where GM10-OEMtLacA-64 reached approximately 4.7-fold of the GM8 biomass level at 1% substrate concentration. These strains also showed increased growth on lignin-derived substrates relative to GM8.

The combination of iterative genome engineering, QPix-assisted colony isolation, and ABTS-based screening allowed rapid comparison of large transformant populations during strain optimization. Among the engineered strains, T64 consistently showed the highest extracellular protein accumulation and laccase activity and was, therefore, selected as the chassis strain for subsequent recombinant protein expression. In this background, the MtLacA locus can be readily replaced by recombinant expression cassettes through CRISPR/Cas9-mediated knock-in, whereas loss of ABTS oxidation activity provides a convenient phenotypic marker for strain screening.

Compared with previous strain engineering approaches in filamentous fungi, our workflow combines iterative genome engineering, automated colony isolation, and ABTS-based counter-screening. This workflow reduces manual colony handling and enables efficient isolation and screening of large transformant populations. It also simplifies the identification of correctly engineered transformants after each round of genome editing. Although this workflow was developed in *M. thermophila*, it should be adaptable to other filamentous fungi that support efficient targeted genome editing and extracellular reporter expression. However, the reporter system and screening conditions may need to be optimized for different fungal species.

### Production of recombinant fungal enzymes in the screening-enabled chassis

**Fig 5:**
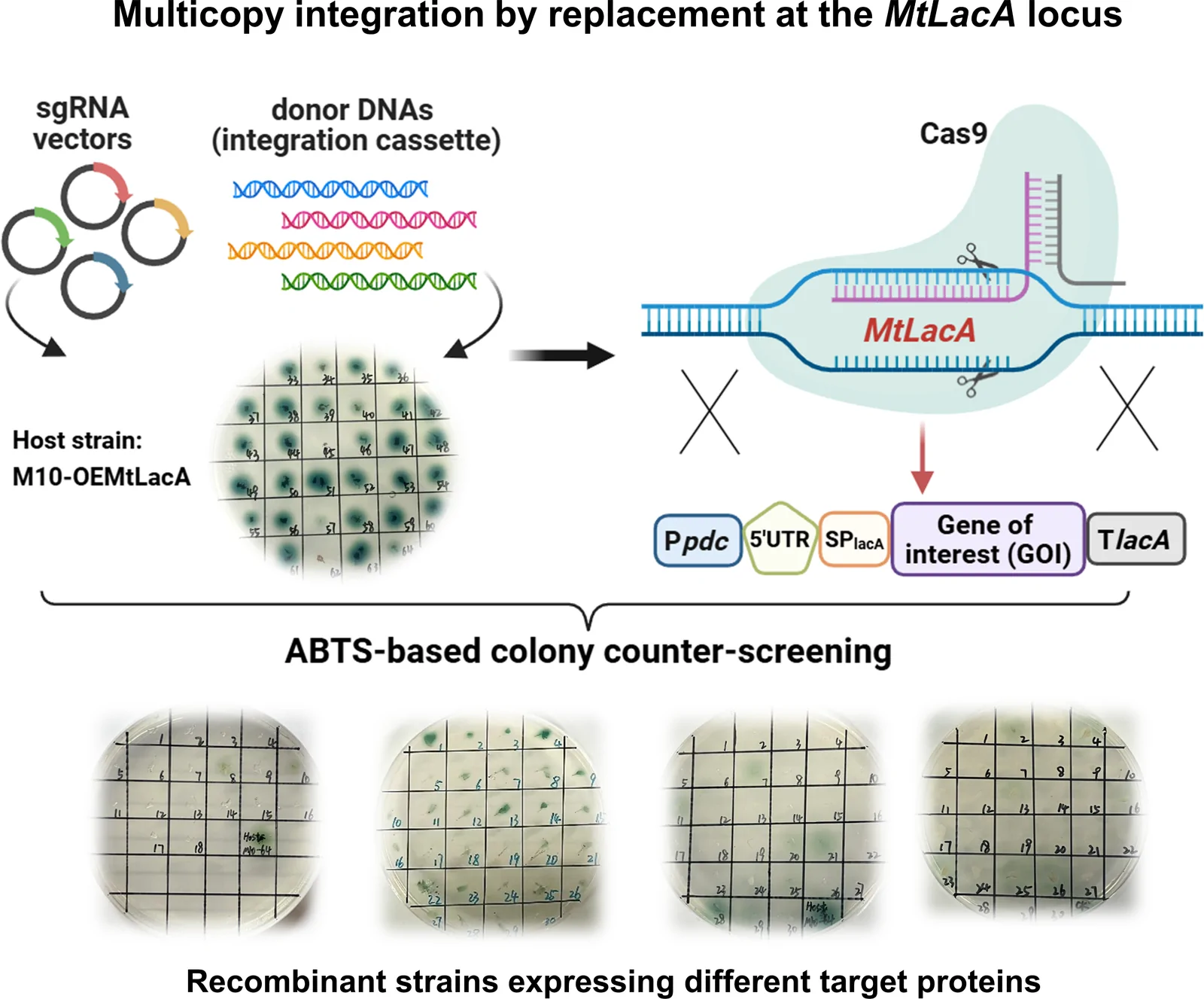
Multicopy integration via replacement of the MtLacA locus and ABTS-based counter-screening of recombinant strains. Schematic illustration of CRISPR-Cas9-mediated replacement of the MtLacA locus with donor DNA cassettes carrying genes of interest (GOIs), enabling multicopy integration in the M10-OEMtLacA host. Disruption of the MtLacA reporter results in loss of laccase activity, allowing recombinant colonies to be distinguished by reduced or absent coloration on ABTS plates. Representative colonies expressing different target proteins are shown.

To further evaluate the applicability of the engineered chassis for heterologous protein production, glucose oxidase from *A. niger* (GoxM) and glucoamylase from *T. emersonii* (TeGlaA) were selected as representative industrial enzymes. As illustrated in Fig. 5, the MtLacA expression cassette in M10-OEMtLacA was replaced with target expression constructs through CRISPR-/Cas9-mediated knock-in. Because replacement of the MtLacA cassette abolished ABTS oxidation activity, recombinant transformants could be readily identified through ABTS-based counter-screening, allowing rapid selection of candidate strains for downstream characterization.

Following counter-screening, representative transformants expressing glucose oxidase (RS-GoxM) and glucoamylase (RS-TeGlaA) were selected for further analysis (Fig. S5). Among the 90 transformants screened for each heterologous protein, 19 RS-GoxM transformants (21.1%) and 20 RS-TeGlaA transformants (22.2%) exhibited positive ABTS-based colony phenotypes. Colony coloration varied markedly among transformants, indicating differences in recombinant protein production. SDS-PAGE analysis confirmed secretion of the target proteins, with protein bands corresponding to the expected molecular weights of GoxM and TeGlaA detected in culture supernatants (Fig. S5). Extracellular protein quantification further demonstrated substantial variation among transformants. The highest-producing RS-GoxM strain reached approximately 521 mg/L extracellular protein, whereas the best-performing RS-TeGlaA strain reached approximately 636 mg/L.

**Fig 6:**
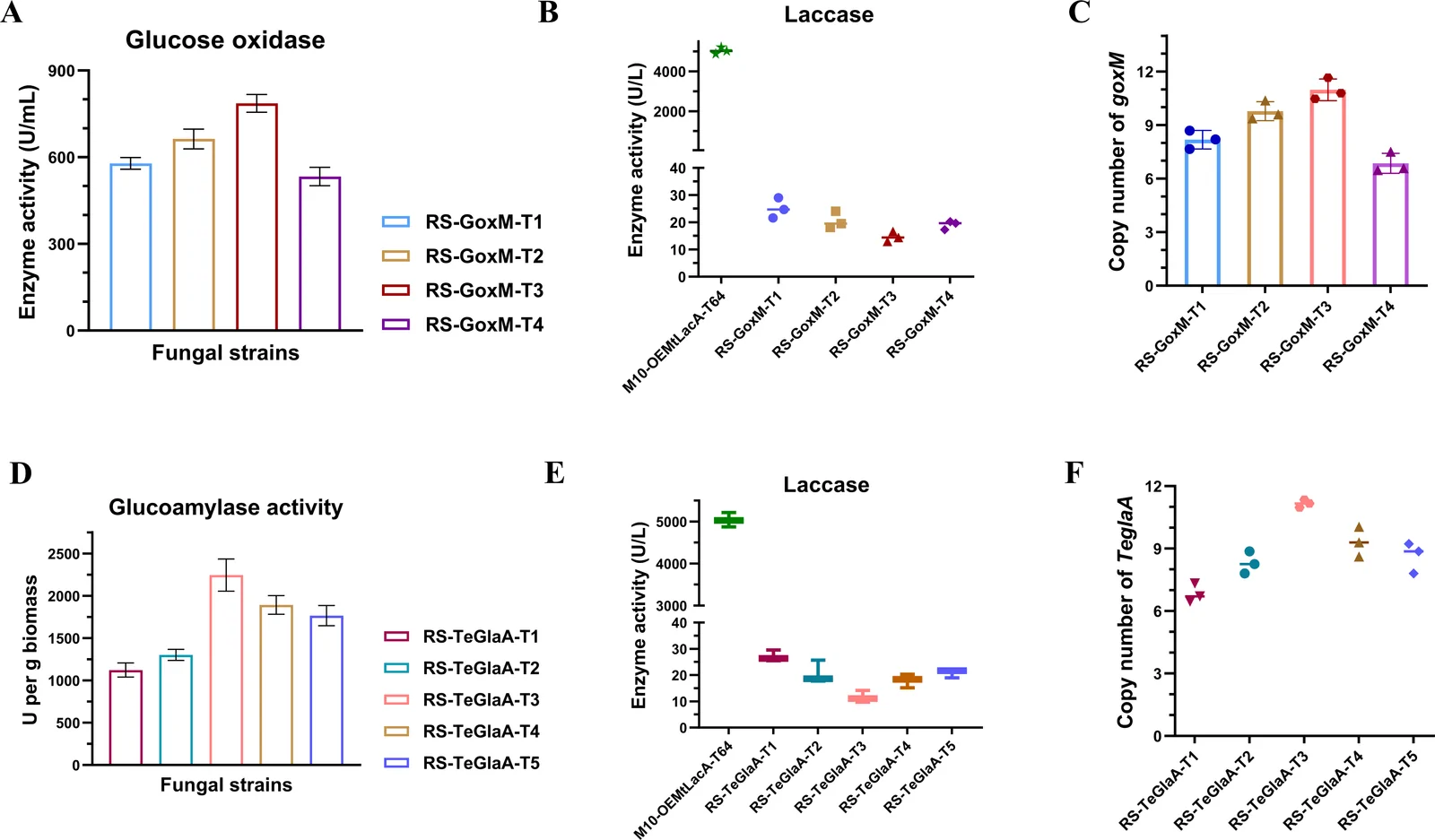
Expression analysis of heterologous fungal enzymes in the engineered chassis. (**A**) Glucose oxidase activity of RS-GoxM transformants. (**B**) Residual laccase activity in RS-GoxM strains compared with the parental strain M10-OEMtLacA-T64. (**C**) Copy number of the *goxM* gene in RS-GoxM transformants determined by qPCR. (**D**) Glucoamylase activity of RS-TeGlaA transformants. (**E**) Residual laccase activity in RS-TeGlaA strains compared with the parental strain. (**F**) Copy number of the TeGlaA gene in RS-TeGlaA transformants determined by qPCR. Enzyme activities were measured from culture supernatants after 6-day shake-flask cultures and are expressed as activity per milliliter of the culture supernatant. Values represent the mean of three independent experiments, with error bars indicating standard deviation.

Representative RS-GoxM transformants identified through ABTS-based colony counter-screening were selected for detailed characterization. Glucose oxidase activity varied substantially among transformants (Fig. 6A). RS-GoxM-T3 exhibited the highest activity (~786 U/mL), followed by RS-GoxM-T2 (~663 U/mL), whereas RS-GoxM-T1 and RS-GoxM-T4 showed comparatively lower activities. Residual laccase activity in all representative RS-GoxM transformants remained close to background levels relative to the parental strain M10-OEMtLacA-T64 (Fig. 6B), consistent with replacement of the MtLacA reporter cassette. Quantitative PCR analysis showed that goxM copy numbers ranged from approximately 7 to 11 among the transformants (Fig. 6C). Although strains carrying higher copy numbers generally exhibited stronger glucose oxidase activity, the relationship was not strictly proportional.

Similarly, representative RS-TeGlaA transformants identified through ABTS-based colony counter-screening were selected for detailed characterization. RS-TeGlaA-T3 exhibited the highest glucoamylase activity (~2,245 U/g biomass), followed by RS-TeGlaA-T4 and RS-TeGlaA-T5, whereas RS-TeGlaA-T1 and RS-TeGlaA-T2 showed comparatively lower activities (Fig. 6D). Residual laccase activity in all representative RS-TeGlaA transformants again remained near background levels relative to the parental strain (Fig. 6E), further supporting successful replacement of the MtLacA reporter cassette. Copy number analysis indicated that TeGlaA was integrated at approximately 7–11 copies among the transformants (Fig. 6F). As observed for GoxM, increased gene dosage was generally associated with higher enzyme activity, although the relationship was not linear.

Despite consistently low residual laccase activity among the engineered strains, glucose oxidase and glucoamylase production varied substantially among transformants. These observations suggest that factors other than gene copy number also influence recombinant protein production and activity. Although enzyme activity generally correlated with gene copy number, noticeable differences were still observed among transformants carrying similar copy numbers. Such variation is common in filamentous fungi and may result from differences in homologous recombination events, local chromatin environment, or transcriptional regulation after integration. In addition, post-transcriptional and post-translational processes may also influence recombinant protein production. These factors may collectively contribute to the variation observed among transformants carrying similar copy numbers at the same genomic locus. Collectively, these results demonstrate that the M10-OEMtLacA chassis supports secretion of heterologous fungal enzymes while enabling straightforward identification of recombinant transformants through ABTS-based counter-screening.

### Production of porcine growth factors and a fusion protein in the engineered fungal chassis

**Fig 7:**
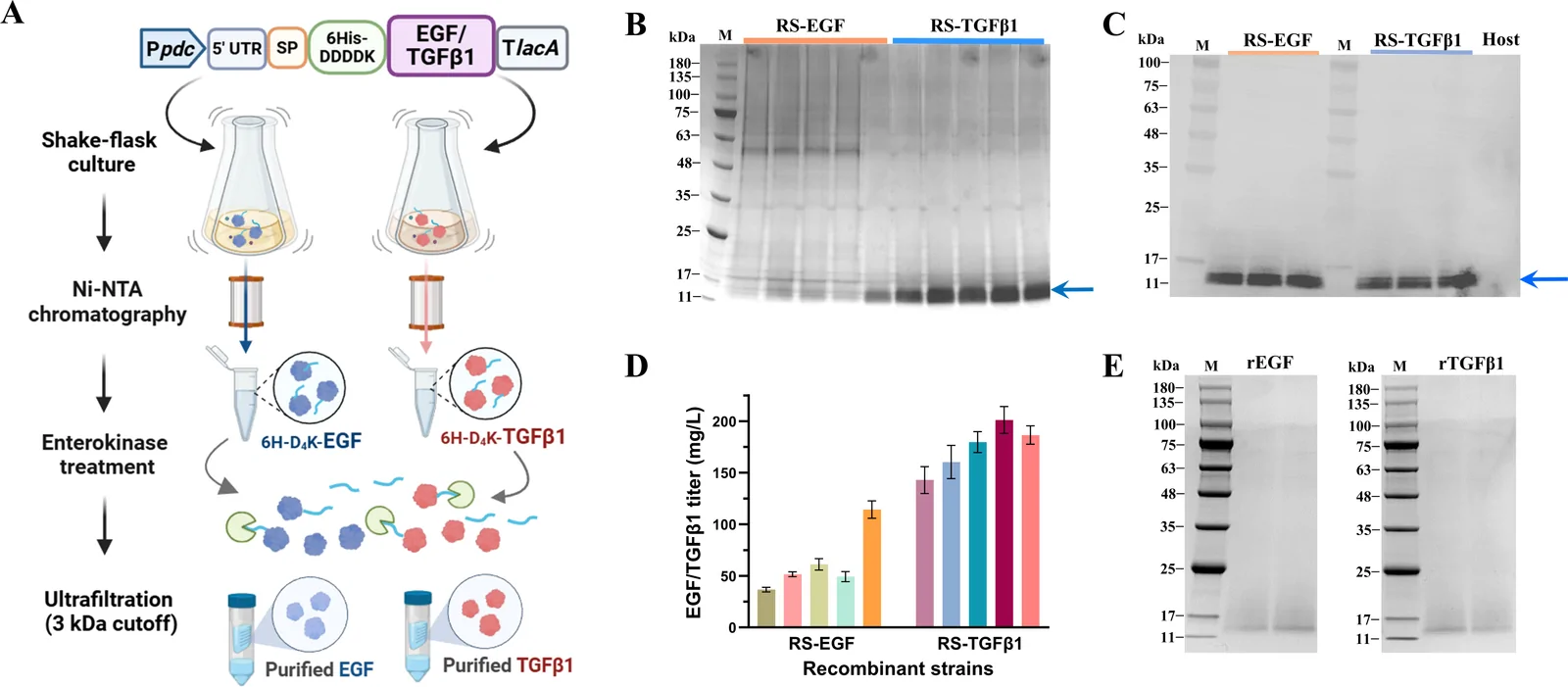
Expression and purification of recombinant growth factors in the engineered chassis. (**A**) Schematic illustration of expression, purification, and processing of EGF and TGFβ1. Proteins were expressed with an N-terminal 6× His–DDDDK tag, purified by Ni-NTA chromatography, treated with enterokinase, and concentrated by ultrafiltration. (**B**) SDS-PAGE analysis of culture supernatants from RS-EGF and RS-TGFβ1 strains after shake-flask cultivation. (**C**) Western blot analysis using anti-His antibodies to confirm the expression of EGF and TGFβ1. The host strain was used as a control. (**D**) Titers of EGF and TGFβ1 in shake-flask cultures. (**E**) SDS-PAGE analysis of purified recombinant EGF (rEGF) and TGFβ1 (rTGFβ1). Values are expressed as means ± SD (*n* = 3 repeats).

**Fig 8:**
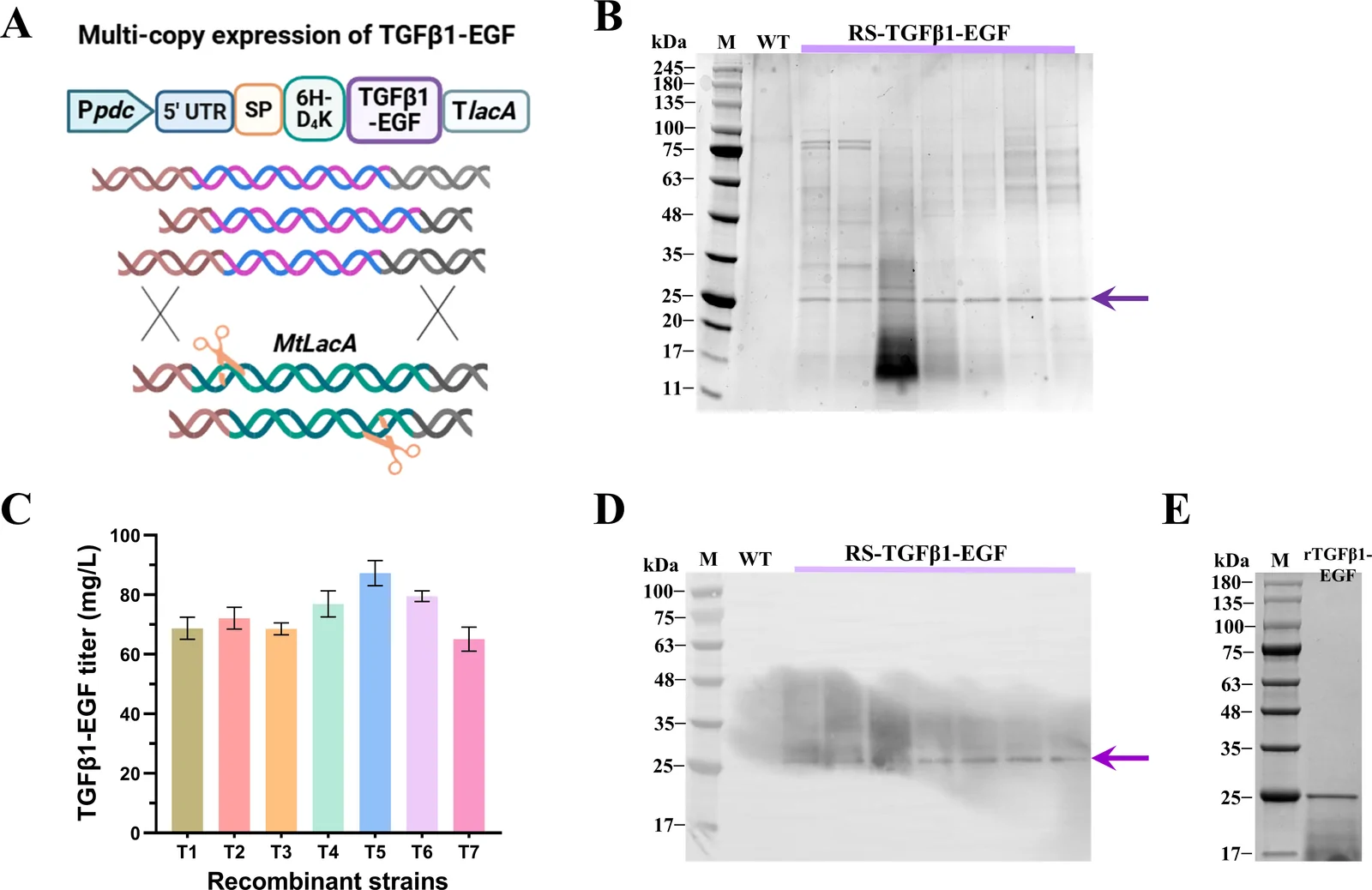
Multicopy expression and characterization of the TGFβ1-EGF fusion protein. (**A**) Schematic illustration of the expression cassette and multicopy integration of the TGFβ1-EGF fusion at the MtLacA locus. (**B**) SDS-PAGE analysis of culture supernatants from RS-TGFβ1-EGF strains. The arrow indicates the fusion protein band. (**C**) Titers of TGFβ1-EGF in recombinant strains cultivated in shake flasks. (**D**) Western blot analysis using anti-His antibodies to confirm the expression of the TGFβ1-EGF fusion protein. (**E**) SDS-PAGE analysis of recombinant TGFβ1-EGF (rTGFβ1-EGF) following Ni-NTA purification and concentration by ultrafiltration. Data are presented as mean ± SD (*n* = 3 biological replicates).

To further evaluate the engineered chassis beyond fungal enzyme production, porcine epidermal growth factor (EGF) and transforming growth factor β1 (TGFβ1) were selected as representative secreted proteins because of their established roles in cell proliferation and tissue development (50, 51). In addition, a TGFβ1-EGF fusion protein was constructed to assess the expression of a more complex recombinant protein (Fig. 7 and 8).

Expression constructs were designed with an N-terminal secretion signal together with a 6× His tag and enterokinase cleavage site to facilitate downstream purification (Fig. 7A). Following shake-flask cultivation, culture supernatants were analyzed by SDS-PAGE and Western blotting. Distinct protein bands corresponding to the expected molecular weights of EGF and TGFβ1 were detected in recombinant strains but were absent in the host control (Fig. 7B and C). After Ni-NTA purification, enterokinase digestion, and ultrafiltration, purified proteins were recovered as discrete bands corresponding to the expected molecular sizes (Fig. 7E), confirming successful secretion and recovery of the recombinant growth factors.

Protein production varied among transformants (Fig. 7D). In RS-EGF strains, recombinant protein titers ranged from approximately 37 to 114 mg/L. RS-TGFβ1 strains showed comparatively higher production, with the best-performing transformants reaching approximately 142–201 mg/L. These results indicate that the engineered chassis supports the secretion of small heterologous growth factors in addition to fungal enzymes.

To further assess system performance using a more complex construct, a TGFβ1-EGF fusion protein was expressed using the same strategy (Fig. 8A). Multiple RS-TGFβ1-EGF transformants were obtained, and SDS-PAGE analysis identified protein bands corresponding to the expected molecular weight of the fusion protein in several strains but not in the host control (Fig. 8B). Recombinant protein titers ranged from approximately 65 to 87 mg/L among the tested transformants (Fig. 8C). After purification, a corresponding protein band was also detected for the fusion construct (Fig. 8E), further confirming successful expression and secretion of the recombinant fusion protein.

Although the biological activities of the recombinant growth factors were not evaluated in this study, the successful recovery of EGF, TGFβ1, and the TGFβ1-EGF fusion protein indicates that the engineered *M. thermophila* chassis is compatible with the secretion of structurally distinct recombinant proteins. These results further support the applicability of the screening-enabled fungal chassis for heterologous protein production.

Compared with conventional manual screening procedures, the semi-automated workflow established in this study increased transformant recovery and screening capacity (Table 1). Traditional screening of filamentous fungal transformants typically relies on manual colony isolation and labor-intensive preliminary evaluation, limiting the number of candidates that can be processed in parallel. In contrast, the combination of QPix-assisted colony picking and ABTS-based activity screening enabled recovery of more than 500 transformants in a single engineering round and parallel evaluation of 96 transformants in deep-well plates. The workflow reduced manual handling and facilitated iterative strain engineering in *M. thermophila*.

**TABLE 1:** Comparison of conventional manual and semi-automated screening workflows

Parameter	Conventional manual workflow	Semi-automated workflow (this study)
Colony isolation	Manual colony picking	QPix-assisted automated picking
Transformant recovery	Limited by manual handling	>500 colonies recovered per engineering round
Screening scale	Typically tens of transformants	96 transformants evaluated in parallel
Primary screening strategy	Random or morphology-based selection	ABTS-based activity screening
Labor intensity	High	Reduced
Applicability to thermophilic fungi	Limited automation reported	Demonstrated in *M. thermophila*

Several engineering and screening strategies have been developed in filamentous fungi. In *T. reesei*, fluorescence-assisted cell sorting (FACS)-based screening and droplet-microfluidic platforms have been applied for the identification and evaluation of cellulase-producing strains (52–54). Similarly, automated biofoundry workflows have been developed in *A. oryzae* for strain construction and pathway characterization (55). In addition, a flow cytometry-based plating-free system was established for direct screening and isolation of transformed protoplasts in *M. thermophila* and *A. niger* (35). These studies illustrate the diverse approaches that have been developed to improve strain construction and screening in filamentous fungi. The reported systems differ in their screening principles and levels of automation, including fluorescence-based sorting, droplet microfluidics, and biofoundry-assisted strain construction. By comparison, the workflow described here combines QPix-assisted colony isolation with activity-based screening in *M. thermophila*. This workflow enabled transformant recovery, parallel strain evaluation, and iterative strain engineering using standard microbiological equipment. Together with the benchmarking results summarized in Table 1, these features establish a semi-automated strain engineering and screening workflow for thermophilic filamentous fungi and provide a practical framework for recombinant protein production in *M. thermophila*.

### Conclusions

This study demonstrates that the M10-OEMtLacA chassis supports the expression and secretion of different recombinant proteins, including fungal enzymes, growth factors, and fusion proteins. Conventional strain engineering and screening in filamentous fungi remain labor-intensive and often require extensive transformant recovery and evaluation. Here, iterative genome engineering combined with QPix-assisted colony picking enabled parallel recovery and screening of transformants in *M. thermophila*. In total, 553 fungal colonies were recovered and processed during a single engineering round using dilution-based colony recovery and QPix-assisted colony isolation, and 96 transformants were further evaluated by ABTS-based plate screening. Targeted replacement at the MtLacA locus, together with ABTS-based counter-screening, provided a convenient phenotypic strategy for identifying candidate recombinants through loss of laccase activity. This approach frequently generated transformants carrying multicopy integrations, typically around 10 copies, and supported recombinant protein production across independent strains. Through iterative screening and strain improvement, extracellular protein production reached approximately 1.1 g/L in shake-flask cultures and 5.9 g/L in a preliminary 5-L fermentation. Although the biological activities of the recombinant growth factors were not examined in this study, successful secretion and recovery after purification indicate that the engineered chassis is compatible with the production of small secreted proteins in filamentous fungi.

Overall, this work combines iterative genome engineering with semi-automated colony isolation and activity-based screening in a thermophilic fungal system. Rather than increasing intrinsic transformation efficiency, the workflow improves transformant recovery, strain evaluation, and screening throughput during fungal strain engineering. The strategy described here may provide a practical reference for developing semi-automated engineering workflows and recombinant protein production systems in other filamentous fungi.

## Data Availability

All of the data generated in this study are available and included in the main text and supplemental material. The raw sequencing data for the resequenced genomes are available in the Sequence Read Archive (SRA) at the National Center for Biotechnology Information (NCBI) under BioProject accession number PRJNA1511484.

## SUPPLEMENTAL MATERIAL

Supplemental material aem.00995-26-s0001.docx Fig. S1 to S6; Table S1.

ASM does not own the copyrights to Supplemental Material that may be linked to, or accessed through, an article. The authors have granted ASM a non-exclusive, world-wide license to publish the Supplemental Material files. Please contact the corresponding author directly for reuse.
