# Supplementary material for: Semi-automated strain engineering and screening in the thermophilic fungus Myceliophthora thermophila for recombinant protein production
Source: Appl Environ Microbiol. 2026 Aug 28;92(9):e00995-26. doi: 10.1128/aem.00995-26 (PMC13599523; doi:10.1128/aem.00995-26)
Supplement: Supplementary file 1 [file aem.00995-26-s0001.docx]

**Supplemental material for Applied and Environmental Microbiology**

**Semi-automated strain engineering and screening in the thermophilic fungus *Myceliophthora thermophila* for recombinant protein production**

Chaohui Gong^1,2,3†^, Xinyu Yang^1,2,3†^, Xiucai Zhao^1,2,3^, Fufan Gou^2,3^, Jie Zhou^2,3^, Wenliang Sun^2,3^, Tao Sun^2,3^, Wenlong Liu^1,4^, Kefen Wang^4^, Xingji Wang^4^, Yefu Chen^1^, Yihan Liu^1^, Chaoyou Xue^2,3^, Qian Liu^2,3*^, Chaoguang Tian^2,3*^

^1^ College of Biotechnology, Tianjin University of Science and Technology, Tianjin 300457, China

^2^ State Key Laboratory of Engineering Biology for Low-Carbon Manufacturing, Tianjin Institute of Industrial Biotechnology, Chinese Academy of Sciences, Tianjin, 300308, China

^3^ National Technology Innovation Center of Synthetic Biology, Tianjin 300308, China

^4^ Longda Biotechnology Inc, Shandong, Linyi 276400, China

^†^Chaohui Gong and Xinyu Yang contributed equally to this work.

*Address correspondence to Qian Liu (liu_q1@tib.cas.cn), or Chaoguang Tian (tian_cg@tib.cas.cn)

**Supplemental information**

**Fig. S1.** CRISPR/Cas9-mediated construction and PCR verification of the GM4 mutant strain. Schematic illustration of targeted gene disruption in the MtWT host using CRISPR/Cas9. Gene replacement was introduced at the *Mtexo-1*, *Mtstk-12*, *Mtspr-14*, and *Mtap3m* loci. PCR analysis confirms successful editing events. Expected band sizes for host and mutant strains are indicated on the right of each gel. Asterisks indicate representative positive transformants.

**Fig. S2.** CRISPR/Cas9-mediated construction and PCR verification of the GM8 mutant strain. Sequential genome editing in the GM4 host targeting the *neo*, *Mtdsc-1*, *Mtsah-2*, *Mtalp-1*, and *Mtdsc-2* loci. PCR analysis confirms the expected gene modifications, with band sizes for host and mutant strains indicated. Asterisks indicate representative positive transformants.

**Fig. S3.** QPix-assisted screening workflow for laccase-producing transformants in *M. thermophila*. Transformants were recovered on screening plates after fungal transformation and dilution-based colony recovery. Colonies were subsequently processed using automated liquid handling and QPix-assisted colony picking, followed by cultivation in deep-well plates. Laccase-producing transformants were identified on ABTS-containing plates by formation of green oxidation halos surrounding fungal colonies.

**Fig. S4.** SDS–PAGE analysis of extracellular proteins from MtLacA overexpression transformants. Extracellular proteins from the host strain and representative transformants (T3–T91) were analyzed by SDS–PAGE after shake-flask cultivation. Variations in overall band intensity among strains reflect differences in protein secretion levels. M, protein marker.

**Fig. S5.** Screening and characterization of fungal enzyme expression in the M10-OEMtLacA chassis. (A) ABTS-based colony counter-screening of glucose oxidase transformants (RS-GoxM). (B) SDS–PAGE analysis of extracellular proteins from representative RS-GoxM transformants (T1–T4); the arrow indicates the GoxM band. (C) Total extracellular protein levels of RS-GoxM transformants determined in shake-flask cultures. (D) ABTS-based colony counter-screening of glucoamylase transformants (RS-TeGlaA). (E) SDS–PAGE analysis of extracellular proteins from representative RS-TeGlaA transformants (T1–T5); the arrow indicates the TeGlaA band. (F) Total extracellular protein levels of RS-TeGlaA transformants determined in shake-flask cultures. Data are shown as mean ± SD from three biological replicates.

**Fig. S6.** Whole-genome sequencing analysis supports amplification of the *MtLacA* gene. (A) Representative IGV views of the reference genomic region containing the *MtLacA* gene (NCBI Reference Sequence: XM_003663693.1) in the parental strain GM8-OEMtLacA-M4 and the engineered strains T8, T9, and T64. Whole-genome sequencing reads were aligned to the reference genome, and copy number variation was analyzed using CNVnator. The duplicated reference region identified by CNVnator and the corresponding normalized read depth (natorRD) are shown for each strain. (B) Normalized read depth of the *MtLacA* reference region estimated by CNVnator from whole-genome sequencing data. The normalized read depth increased progressively from GM8-OEMtLacA-M4 to T8, T9, and T64, consistent with the qPCR-estimated increase in *MtLacA* copy number.

**Table S1.** List of PCR primers used in this study.

**Fig. S1.** CRISPR/Cas9-mediated construction and PCR verification of the GM4 mutant strain. Schematic illustration of targeted gene disruption in the MtWT host using CRISPR/Cas9. Gene replacement was introduced at the *Mtexo-1*, *Mtstk-12*, *Mtspr-14*, and *Mtap3m* loci. PCR analysis confirms successful editing events. Expected band sizes for host and mutant strains are indicated on the right of each gel. Asterisks indicate representative positive transformants.

**Fig. S2.** CRISPR/Cas9-mediated construction and PCR verification of the GM8 mutant strain. Sequential genome editing in the GM4 host targeting the *neo*, *Mtdsc-1*, *Mtsah-2*, *Mtalp-1*, and *Mtdsc-2* loci. PCR analysis confirms the expected gene modifications, with band sizes for host and mutant strains indicated. Asterisks indicate representative positive transformants.

**Fig. S3.** QPix-assisted screening workflow for laccase-producing transformants in *M. thermophila*. Transformants were recovered on screening plates after fungal transformation and dilution-based colony recovery. Colonies were subsequently processed using automated liquid handling and QPix-assisted colony picking, followed by cultivation in deep-well plates. Laccase-producing transformants were identified on ABTS-containing plates by formation of green oxidation halos surrounding fungal colonies.

**Fig. S4.** SDS–PAGE analysis of extracellular proteins from MtLacA overexpression transformants. Extracellular proteins from the host strain and representative transformants (T3–T91) were analyzed by SDS–PAGE after shake-flask cultivation. Variations in overall band intensity among strains reflect differences in protein secretion levels. M, protein marker.

**Fig. S5.** Screening and characterization of fungal enzyme expression in the M10-OEMtLacA chassis. (A) ABTS-based colony counter-screening of glucose oxidase transformants (RS-GoxM). (B) SDS–PAGE analysis of extracellular proteins from representative RS-GoxM transformants (T1–T4); the arrow indicates the GoxM band. (C) Total extracellular protein levels of RS-GoxM transformants determined in shake-flask cultures. (D) ABTS-based colony counter-screening of glucoamylase transformants (RS-TeGlaA). (E) SDS–PAGE analysis of extracellular proteins from representative RS-TeGlaA transformants (T1–T5); the arrow indicates the TeGlaA band. (F) Total extracellular protein levels of RS-TeGlaA transformants determined in shake-flask cultures. Data are shown as mean ± SD from three biological replicates.

**Fig. S6.** Whole-genome sequencing analysis supports amplification of the *MtLacA* gene. (A) Representative IGV views of the reference genomic region containing the *MtLacA* gene (NCBI Reference Sequence: XM_003663693.1) in the parental strain GM8-OEMtLacA-M4 and the engineered strains T8, T9, and T64. Whole-genome sequencing reads were aligned to the reference genome, and copy number variation was analyzed using CNVnator. The duplication identified by CNVnator within the reference region and the corresponding normalized read depth (natorRD) are shown for each strain. (B) Normalized read depth of the *MtLacA* reference region estimated by CNVnator from whole-genome sequencing data. The normalized read depth increased progressively from GM8-OEMtLacA-M4 to T8, T9, and T64, consistent with the qPCR-estimated increase in *MtLacA* copy number.

**Table S1.** List of PCR primers used in this study.

| **Primer name** | **Primer Sequence (5' to 3')** |
| --- | --- |
| Ppdc-F | TGAAGTAATCTCTGCAGATCTTTAATT |
| Ppdc-R | AGGGGCAGCAGCAACGCTAGGGGTGAG |
| lacA-F | ACTAGTATGAAGTCCTTCATCAGCGCCGCGACG |
| lacA-R | TCGCGGATCCTCAGTGGTGGTGGTGGTGGTGGATATCCGCCTTGACCAGCCACTCGCCCTCC |
| TlacA-F | CGGATATCCACCACCACCACCACCACTGAGGATCCGCGAGGGAGGAAAAAGGAAACAAAG |
| TlacA-R | AAGTGCTCCTTCAATATCAGTTAACGTCGTCTAGAGCAGTCCTTGCAGATACTGCGCTGG |
| Ppdc-SF | AGATATGTGTCACCACTCAAGACCC |
| TlacA-SR | TATTTACCCGATCACACACCCACTC |
| TeGlaA-F | TCATCAACACATCATCAAGACACCACCGCGGCCGCATGGCGTCCCTCGTTGCTGGCGCT |
| TeGlaA-R | CTCTTTGTTTCCTTTTTCCTCCCTCGCGGATCCTCAGTGGTGGTGGTGGTGGTGGTGGT |
| GoxM-F | TCATCAACACATCATCAAGACACCACCGCGGCCGCATGGTCGCGTGGTGGTCTCTATTT |
| GoxM-R | CCTCTTTGTTTCCTTTTTCCTCCCTCGCGGATCCTTAGTGGTGGTGGTGGTGGTGTTGC |
| GF-F | AATCATCAACACATCATCAAGACACCACCGCGGCCGCT |
| GF-R | CCCCTCTTTGTTTCCTTTTT CCTCCCTCGCGGATCCTTA |
| pCas9-F | TCCTCCGAGGTTCGACATCAGGGTT |
| pCas9-R | CCTCTAAACAAGTGTACCTGTGCAT |
| U6p-F | AGGATCGGTGGAGTGAAGTTCGGAA |
| gRNA-R | AAAAAAAGCACCGACTCGGTGCCACTT |
| U6_lacT1-R | GCTCTAAAACCTCCGTGATCGGGACGAGCACGAGGAAAGAAAGAAAAGAAGAGGAG |
| gRNA_lacT1-F | TTCTTTCCTCGTGCTCGTCCCGATCACGGAGGTTTTAGAGCTAGAAATAGCAAGTT |
| U6_lacT2-R | GCTCTAAAACCTTCGTAGTCGGTGTTGATGTCGAGGAAAGAAAGAAAAGAAGAGGAG |
| gRNA_lacT2-F | TTCTTTCCTCGACATCAACACCGACTACGAAGGTTTTAGAGCTAGAAATAGCAAGTT |
| Mtalp1-ko-F | CCTCGCAGCAAACTCGAGCTGCTAT |
| Mtalp1-ko-R | CGGCGTTGTTGATGGAGGCCGAGTA |
| Mtspr14-ko-F | TGTCCATCCGGTTGAAGAGTGGGT |
| Mtspr14-ko-R | TGGACATCGGCGCCCTTGACGACAT |
| neo-ko-F | AACCCCAGGCATCGTAGATCAGGGC |
| neo-ko-R | GAGAAGTGGGACTGGTACGAGACAA |
| Mtexo1-ko-F | GTCCTGTTTCAGCCCATCGTCGCAA |
| Mtexo1-ko-R | CTTGAGGTGCAGCTTTCGGTATGTT |
| bar-ko-F | TTTGGCACTCGTGACCAGTCTGCAA |
| bar-ko-R | TAGCCGCTGAACTGCTTGACCGAAT |
| Mtres1-ko-F | GCGACTACCTCCCCGGCGGCGAATA |
| Mtres1-ko-R | GCATATGAGCCTTGAGGTCCTTGAA |
| Mtap3m-ko-F | CTGTTCCGCGCCCGAACCTCATCTA |
| Mtap3m-ko-R | GTTGTAGGTGGCGTCCCCCTTTGTA |
| Mtstk12-ko-F | TTCCAGTGTCGCTACCGATGGGCTT |
| Mtstk12-ko-R | CCGGATCATCCACATCGTCTGAGAA |
| Mtdsc1-ko-F | CGGGACAACCCAAACGCGACTTGAT |
| Mtdsc1-ko-R | CAGCGCGGCACTGGAACAGAGACTT |
| Mtsah2-ko-F | TAACCTGATTATGTCCGAGTTCCCA |
| Mtsah2-ko-R | TGCGGAGGCATGGCAATGGATTTGA |
| lacA-qF | TACCGCCGCCACAAACGACCCGAAT |
| lacA-qR | AAGATTGTTGGTCCTATG |
| Actin-qF | TGCCCATCTACGAGGGTTTC |
| Actin-qR | TTGATGTCACGGACAATTTCAC |
| EGF-qF | CTTCATCAACACCACCAT |
| EGF-qR | GAGTAGGAGTTCATCTTCTT |
| TGFβ1-qF | CAGCTCTACATCGACTTC |
| TGFβ1-qR | GAGGACCTTGGAGTATTG |
| TeglaA-qF | GTATGCGAACTATCTCATC |
| TeglaA-qR | TTCTACTTCTTCCCAGAG |
| AngoxA-qF | GAACTGTTGACTACATCAT |
| AngoxA-qR | ACCTCTATCAGATTCGTAA |
